# Supplementary material for: Integration of Heterogeneous Biological Data in Multiscale Mechanistic Model Calibration: Application to Lung Adenocarcinoma
Source: Acta Biotheor. 2022 Jul 7;70(3):19. doi: 10.1007/s10441-022-09445-3 (PMC9261258; doi:10.1007/s10441-022-09445-3)
Supplement: Supplementary file 1 — Supplementary material 1 (PDF 139 kb) [file 10441_2022_9445_MOESM1_ESM.pdf]

Integration of heterogeneous biological data in multiscale mechanistic model calibration: app

Supplementary data

Table S1 Summary of parameters to calibrate, the phenomena they belong to and their values evolution over the course of the calibration process.

Parameter description	Phenomena related to	Value prior to calibration (unit)	Value after calibration (unit)
Maximal depth of the tumor at which cells can survive without neoangiogenesis	tumor cell proliferation, tumor cell death	104 (µm)	111.8 (µm)
Ratio of living cells over proliferating ones with the tumor	tumor cell proliferation	5 (unitless)	4.52 (unitless)
Growth rate of the tumor	tumor cell proliferation	0.07 (day^{-1})	0.0751 (day^{-1})
Transduction of EGFR signaling into growth signaling, first parameter: equivalent to the signal required to reach half the maximal growth	tumor cell proliferation	0.6 (unitless)	0.416 (unitless)
Transduction of EGFR signaling into growth signaling, second parameter: equivalent to the sensitivity of tumor growth to change in EGFR signal	tumor cell proliferation	10 (unitless)	2.95 (unitless)
Death rate of the hypoxic tumor due to necrosis	tumor cell death	1 (day^{-1})	0.435 (day^{-1})
Transduction of EGFR signaling into cell survival signaling, first parameter: equivalent to the signal required to reach half the maximal growth	tumor cell death	0.07 (unitless)	0.0439 (unitless)
Transduction of EGFR signaling into cell survival signaling, second parameter: equivalent to the sensitivity of the cell survival to change in EGFR signal	tumor cell death	10 (unitless)	26.3 (unitless)
Maximal cell death inhibition the EGFR cell survival signaling can provide	tumor cell death	0.8 (unitless)	0.743 (unitless)
Number of cells neoangiogenesis can supply within the tumoR	neoangiogenesis	7.15e9 (cells)	2.77e11 (cells)
Immune system cell killing, first parameter: maximal speed of tumor cell killing by the immune system	effect of the immune system on the tumor	3.40e7 ($cells.day^{-1}$)	5.94e9 ($cells.day^{-1}$)
Immune system cell killing, second parameter: affinity tumor cell killing by the immune system	effect of the immune system on the tumor	1e9 (cells)	9.89e10 (cells)
Immune system cell killing, third parameter: maximal number of cells the immune system can have access to	effect of the immune system on the tumor	1e7 (cells)	2.46e9 (cells)
Fraction of unbound gefitinib drug within the tumor	treatment	1 (unitless)	0.401 (unitless)
